# Safety evaluation method of bottom coal thickness in thick coal seam roadway

**DOI:** 10.1038/s41598-024-65708-8

**Published:** 2024-06-27

**Authors:** Yunliang Tan, Shilin Song, Xiufeng Zhang, Xuesheng Liu

**Affiliations:** 1 College of Energy and Mining Engineering, Shandong University of Science and Technology, Qingdao, 266590 China; 2 State Key Laboratory of Mining Disaster Prevention and Control, Shandong University of Science and Technology, Qingdao, 266590 China; 3 Shandong Energy Grp Co Ltd, Jinan, 250101 China

**Keywords:** The floor strength, The bottom coal thickness, Safety evaluation, Energy science and technology, Engineering

## Abstract

In recent years, the number of roadway floor rock burst accidents is increasing, which seriously restricts the safe production of the mine. Therefore, safety evaluation method of bottom coal thickness in thick coal seam roadway was studied. The research results shown that the stress concentration area of composite floor is distributed in coal seam or rock stratum with large elastic modulus. With the increase of floor rock strength, the stress of coal-rock composite floor increased gradually, but the displacement and energy decreased gradually. When floor rock strength was equal to bottom coal strength, the increase of floor stress and displacement with the change of bottom coal thickness was the smallest, which was 34.29% and 33.61% respectively. The elastic strain energy decreased from 14.58 to 9.85%. With the increase of bottom coal thickness, the stress and displacement of coal-rock composite floor increased first and then decreased, and the elastic strain energy decreased gradually. It puts forward the safety evaluation method of bottom coal thickness: floor failure depth → reasonable thickness of bottom coal → safety thickness of bottom coal. It can provide reference for design of roadway bottom coal retention and surrounding rock control in thick coal seam face.

## Introduction

Coal resources have played a crucial role in China's economic development, and they are an important guarantee of China's national strength and the basis of economic progress^1–4^. In recent years, the number of rock burst accidents in roadway floor has been gradually increasing, which mostly occurs in the roadway of super-thick coal seam mining or thick coal seam mining, especially in the roadway with bottom coal^5–8^. It is mainly manifested as instantaneous bulging and cracking of the floor, coal block ejection, equipment dumping and so on. It may be accompanied by a certain degree of damage to the side and roof, causing a large amount of economic losses and casualties, which seriously restricts the safety production of coal mines^9–13^. The key to prevent floor rock burst is not only to study the mechanism of floor rock burst, but also to study the influencing factors of floor rock burst.

At present, due to the limitations of thick coal seam mining technology and actual production geological conditions, such as soft coal body, low hardness, immediate floor and other problems, this kind of roadway must be left with bottom coal driving. However, leaving the bottom coal will inevitably increase the risk of rock burst in the roadway. Therefore, the retention of coal at the bottom of the roadway is an important factor affecting floor rock burst. At the same time, the factors affecting the retention of bottom coal are also complex and diverse, including the thickness of bottom coal, the strength of bottom coal and the strength of floor^14–17^.

It is of great practical significance to study the mechanism and prevention of floor rock burst by mastering the law of influence of floor coal on floor rock burst. Experts at home and abroad have carried out a lot of research work on this. Jiang Fuxing et al. classified floor impact into two types: buckling instability impact of rock floor and dilatancy impact of thick bottom coal^18^. Xu Xuefeng et al. established a mechanical model of the occurrence conditions and influencing factors of floor rock burst, and proposed a calculation method for the risk coefficient of floor rock burst^19^. Xie Long et al. established the stress model of roadway in the tectonic stress field, and obtained the influence law of lateral pressure coefficient on roadway floor impact in extremely thick coal seam induced by dynamic load^20^. Shi Qingwen believed that the thickness of the bottom coal, the hardness of the coal and rock mass of the floor, the lateral abutment pressure of the two sides, and the dynamic load strength were the main factors that induced the floor rock burst^21^. Zhang Chenyang et al. believed that the thickness of bottom coal was a key factor affecting the storage of elastic energy in bottom coal. The thickness of bottom coal affected the risk of the floor impact by affecting the transfer of elastic energy between bottom coal and bottom rock^22,23^. Wang Xuhong et al. pointed out that with the increase of roadway width, the maximum horizontal tensile stress gradually developed to the depth of floor. And it would result in sudden fracture of key floor layers and induce floor rock burst^24^. Through numerical simulation, Chen Feng et al. believed that dynamic disturbance was a major factor to break the limit equilibrium state of coal rock mass systems^25^. Xiao Zhimin et al., Li Baofu et al., believed that the horizontal stress of roadway floor was the main inducing factor of floor impact^26,27^. Gai Decheng et al. found that the maximum range of floor slip impact is related to the width of coal pillar and the internal friction angle of floor strata^28^. The above research provides the strong support for the mechanism research and prevention technology of floor rock burst.

At once, the evaluation studies based on thick coal seam mining are mostly risk assessment of rock burst^29,30^, coal pillar stability evaluation^31,32^ and roof safety evaluation^33,34^^.^ The above research provides strong support and guidance for the safe mining of thick coal seam face, but there are few studies on the safety evaluation of bottom coal roadway in thick coal seam. Therefore, the safety evaluation of bottom coal thickness still needs to be further carried out.

At present, there are still deficiencies in the research on the safety thickness of bottom coal in rock burst mine. For example, the relationship between the strength of floor and the thickness of bottom coal, the influence of the strength of floor on the coal retention of floor, etc. In the existing studies, most experts and scholars only study a certain roadway, without considering the influence of mining near the working face or the working face being mined. This manuscript took the thick coal seam face as the geological background, and adopted theoretical analysis and numerical simulation method. The safety evaluation method of bottom coal thickness in thick coal seam roadway is put forward. It provides a basis for safe mining of thick coal seam face.

## Mechanical analysis of floor failure mechanism and floor failure depth of bottom coal roadway

### Failure mechanism and analysis of roadway floor

Under the influence of the lateral abutment pressure of the adjacent goaf, the surrounding rock of the mining roadway is broken. The broken surrounding rock can be regarded as a loose body with poor bearing capacity. At the same time, considering the bottom coal left in the floor during the mining of thick coal seam, the failure mechanism of such roadway floor is analyzed according to the theory of material mechanics.

**Figure 1:**
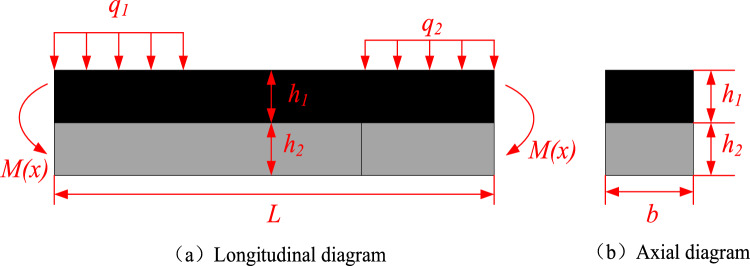
Mechanical model of floor coal-rock composite beam.

When the impact instability occurs in the bottom coal roadway, the bottom coal and the floor undergo flexural deformation under the action of deflection. The flexural failure cracks of the floor are generally generated from the central position of the roadway and extend to the side of the roadway. Regardless of the water swelling and rheological properties of the floor rock, it is assumed that the bottom coal and the floor rock have the same flexural center and the flexural deformation curve is also similar, and the two are in a long-term compaction state. Therefore, the bottom coal and the floor rock can be regarded as a composite beam structure to establish a mechanical model, as shown in Fig. 1.

As shown in Fig. 1, the section bending moment *M(x)* of the coal-rock composite beam was generated by the action of external load, so the following formula should be established:

1 $$M\left(x\right)={M}_{1}\left(x\right)+{M}_{2}(x)$$

where *M(x)* is the bending moment of the whole beam section acting on the coal-rock composite beam; *M*_*1*_*(x)* is the bending moment of the beam section acting on the bottom coal; *M*_*2*_*(x)* is the bending moment of the beam section acting on the floor rock.

Assuming that the contact surface of the two beams is smooth, the cross section of the composite beam is rotated around the respective neutral axis when it is bent. Under the condition of small deformation, the curvature of the two beams is equal everywhere, and they bear the mid-span bending moment together. Thus, the mid-span section has:

2 $$M={M}_{1}+{M}_{2}$$

3 $$\frac{1}{{\rho }_{1}}=\frac{1}{{\rho }_{2}}$$

And because of the following formula:

4 $$\frac{1}{{\rho_{1} }} = \frac{{M_{1} }}{{E_{1} I_{z1} }},\;\frac{1}{{\rho_{2} }} = \frac{{M_{2} }}{{E_{2} I_{z2} }}$$

where 1/*ρ* is the curvature of the composite beam; *E* is Stiffness of the composite beam; *I*_*z*_ is inertia moment of composite beam.

It can be obtained:

5 $$M_{1} = \frac{1}{{1 + E_{2} I_{z2} /E_{1} I_{z1} }}M,\;M_{2} = \frac{1}{{1 + E_{1} I_{z1} /E_{2} I_{z2} }}M$$

The maximum normal stress of the upper and lower beams is:

6 $$\sigma_{1,\max } = \frac{{M_{1} }}{{I_{1} }}\frac{{h_{1} }}{2} = \frac{1}{{1 + E_{2} I_{z2} /E_{1} I_{z1} }}\frac{M}{{I_{z1} }}\frac{{h_{1} }}{2}$$

7 $$\sigma_{2,\max } = \frac{{M_{2} }}{{I_{2} }}\frac{{h_{2} }}{2} = \frac{1}{{1 + E_{1} I_{z1} /E_{2} I_{z2} }}\frac{M}{{I_{z2} }}\frac{{h_{2} }}{2}$$

where *σ* is the normal stress of composite beam; *h* is the height of the upper and lower beams.

It is assumed that when the composite beam is bent, the cross section remains plane, just like the monolithic beam. According to the condition that the axial force on the section is zero, the formula for determining the neutral axis can be obtained:

8 $${E}_{1}{S}_{z1}+{E}_{2}{S}_{z2}=0$$

where *S*_*z1*_、*S*_*z2*_ is the static moment of the upper and lower sections to the neutral axis.

Assuming that the neutral axis above the composite surface is *y*_*c*_, then there is:

9 $${S}_{z1}=\underset{{A}_{1}}{\overset{\phantom{a}}{\int }}ydA={y}_{c1}{A}_{1}=\left({h}_{1}/2-{y}_{c}\right)b{h}_{1}$$

10 $${S}_{z2}=\underset{{A}_{2}}{\overset{\phantom{a}}{\int }}ydA={-y}_{c2}{A}_{2}=-\left({h}_{2}/2+{y}_{c}\right)b{h}_{2}$$

where *A* is the cross-sectional area of the composite beam.

Bring Eqs. (8) and (9) into Eq. (7), and get:

11 $${y}_{c}=\frac{{E}_{1}{h}_{1}^{2}-{E}_{2}{h}_{2}^{2}}{2\left({E}_{1}{h}_{1}+{E}_{2}{h}_{2}\right)}$$

According to the theory of material mechanics, the calculation formula of the normal stress of the mid-span section is:

12 $${\sigma }_{c}=\frac{M{y}_{c}}{{I}_{z}}$$

The cross section of the composite beam is generally rectangular. Therefore, *I*_*z*_ can be calculated by the following formula:

13 $${I}_{z}=\frac{b{\left({h}_{1}+{h}_{2}\right)}^{3}}{12}$$

To bring Eqs. (2), (4), (10), (12) into Eq. (11), it can get:

14 $${\sigma }_{c}=\frac{M{y}_{c}}{{I}_{z}}=\frac{M\frac{{E}_{1}{h}_{1}^{2}-{E}_{2}{h}_{2}^{2}}{2\left({E}_{1}{h}_{1}+{E}_{2}{h}_{2}\right)}}{b{\left({h}_{1}+{h}_{2}\right)}^{3}/12}$$

15 $$M=\frac{{\sigma }_{c}b{\left({h}_{1}+{h}_{2}\right)}^{3}\left({E}_{1}{h}_{1}+{E}_{2}{h}_{2}\right)}{6\left({E}_{1}{h}_{1}^{2}-{E}_{2}{h}_{2}^{2}\right)}$$

It can be seen from Eq. (15) that the bending moment of the composite beam is related to the normal stress, the stiffness and thickness of the upper and lower beams.

### Failure depth of roadway floor and bottom coal

**Figure 2:**
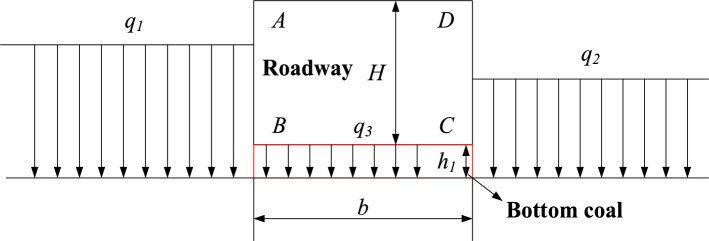
Roadway floor load diagram.

Taking the return roadway of a coal face as an example, one side of the roadway floor is affected by the coal seam pressure of the coal face, and the other side is affected by the coal pillar pressure. In addition, it will also be subject to the pressure generated by the retention of bottom coal. In order to simplify the calculation, it is assumed that the floor of the roadway is subjected to uniform loads of different sizes *q*_*1*_, *q*_*2*_, and* q*_*3*_. As shown in Fig. 2. The roadway floor is damaged under uniform load.

**Figure 3:**
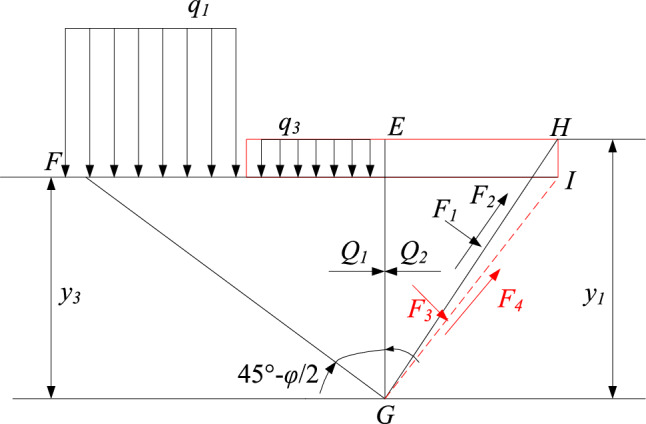
The stress state diagram of the left floor under load.

Taking the left floor as the analysis object, under the combined action of uniform load *q*_*1*_ and *q*_*3*_, the surrounding rock of EFG area and BGH area is in the state of active and passive plastic stress respectively, resulting in upward stress. When the stress reaches the ultimate strength of the combination of the floor rock and the bottom coal, the floor EH is destroyed, and the floor heaving is formed by upward extrusion, and its stress state is shown in Fig. 3.

When the roadway reaches the limit equilibrium, the angle between the slip surface GH and the horizontal line is (45° − *φ*/2), and the angle between the slip surface FG and the horizontal line is (45° + *φ*/2). *φ* is the friction angle of loose rock mass.

From the theory of rock mechanics, it can be known that the active and passive pressure of each point of EG is:

16 $${\sigma }_{a}=\left({q}_{1}+{q}_{3}+\gamma y\right){K}_{a}$$

17 $${\sigma }_{p}=\gamma y{K}_{p}$$

where *γ* is the average bulk density of overlying rock; *q*_*1*_, *q*_*3*_ is uniform load; *y* is the depth of any point on the floor; *K*_*a*_ is active pressure coefficient, *K*_*a*_ = tan^2^(45°—*φ*/2); *K*_*p*_ is passive pressure coefficient,* K*_*p*_ = tan^2^(45° + *φ*/2).

At the G point,* σ*_*p*_ = *σ*_*a*_, the floor is in the limit equilibrium state. Above the G point, *σ*_*p*_ < *σ*_*a*_, the floor is in plastic state. Below the G point, *σ*_*p*_ > *σ*_*a*_, the floor is in elastic state.

When *σ*_*p*_ = *σ*_*a*_, the ultimate failure depth of the left floor is:

18 $${y}_{1}=\frac{{q}_{1}+{q}_{3}}{\gamma }\frac{{K}_{a}}{{K}_{p}-{K}_{a}}$$

When the stress at the G point reaches the limit equilibrium, the upper strata of the roadway floor will uplift upward, and the lower strata will not displace.

If there is no bottom coal at the roadway floor, that is, *q*_*3*_ = 0, the ultimate failure depth of the left floor is:

19 $${y}_{3}=\frac{{q}_{1}}{\gamma }\frac{{K}_{a}}{{K}_{p}-{K}_{a}}$$

Therefore, by subtracting Eq. (16) from Eq. (17), the damage depth of bottom coal can be obtained as follows:

20 $${h}_{3}={y}_{1}-{y}_{3}=\frac{{q}_{3}}{\gamma }\frac{{K}_{a}}{{K}_{p}-{K}_{a}}$$

It is necessary to consider a certain safety factor *K* when setting the bottom coal in thick coal seam roadway. Therefore, the safe thickness of the bottom coal is:

21 $${h}_{4}=K\frac{{q}_{3}}{\gamma }\frac{{K}_{a}}{{K}_{p}-{K}_{a}}$$

**Figure 4:**
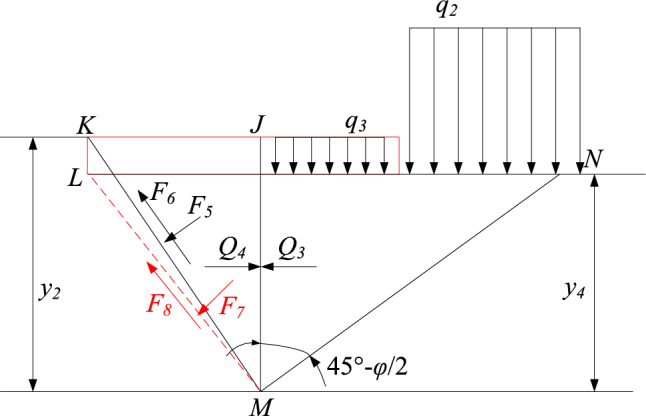
The stress state diagram of the right floor under load.

Similarly, the stress state of the right floor is shown in Fig. 4, and the ultimate failure depth on the right side of the roadway is:

22 $${y}_{2}=\frac{{q}_{2}+{q}_{3}}{\gamma }\frac{{K}_{a}}{{K}_{p}-{K}_{a}}$$

### Example verification

According to the physical and mechanical parameters of different floor lithology given in the ‘MT 553-1996 Classification standard of working face floor in gently inclined coal seam’^35,36^, the physical and mechanical parameters are brought into Eq. (20). The calculation results are shown in Table 1.

**Table 1:** Calculation results of bottom coal thickness under different floor lithology.

Floor strength	*σ*_*RC*_ < *σ*_*MC*_	*σ*_*RC*_ = *σ*_*MC*_	*σ*_*RC*_ > *σ*_*MC*_
Floor failure depth *h*_*3*_/m	0.684	0.658	0.644
Bottom coal safety thickness *h*_*4*_/m	1.026	0.987	0.966

It can be seen from the calculation results of Eq. (20) that the ultimate failure depth of bottom coal under different floor rock strength is less than 1 m. Moreover, with the gradual increase of the strength of the roadway floor rock, the ultimate failure depth of the bottom coal is gradually reduced, but the decrease is not large, which is reduced by 8.3%. In order to ensure the safe and stable use of the roadway, a certain safety factor is usually considered. Therefore, a certain safety factor *K* (*K* = 1.5) is substituted into Eq. (21), and the safe thickness of bottom coal is calculated as shown in Table 1. According to the calculation results of Eq. (21), it can be found that the safety thickness of bottom coal in roadway under three kinds of floor rock strength is about 1 m.

## Numerical simulation study on the thickness of bottom coal in thick coal seam roadway

### Numerical simulation model establishment and scheme

#### Engineering geological conditions

The no. 8302 working face of Xinjulong Coal Mine is the second working face in the eighth mining area of the second level. The coal face is located in the monoclinic structure area of Bigai anticline and Huangtang syncline structure. The dip length is 270 ~ 278 m, the strike length is 1180 m, the buried depth is about 900 m, the average dip angle of coal seam is 5°, and the average coal thickness is 8.97 m.

#### Establishment of numerical simulation model

**Figure 5:**
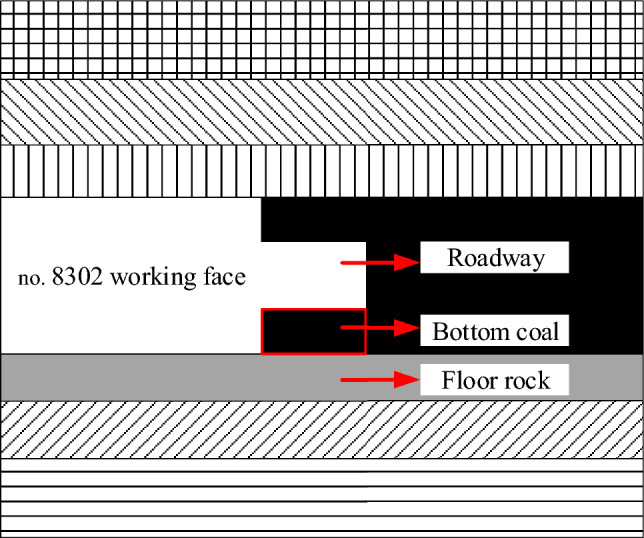
Simplified numerical simulation model.

Based on the geological data of no. 8302 coal face, a numerical simulation model was established. The size of the model was 400 m × 200 m × 150 m, with a total of 4,000,000 nodes and 390,000 grids. The thickness of the coal seam was 8 m, the size of the chamber was 3 m × 4 m, the length of the working face was 240 m, and the mining height was 8 m. The simplified model of numerical simulation was shown in Fig. 5.

The boundary conditions of the model were that X direction constrained the left and right boundary displacement, Y direction constrained the front and back boundary displacement, Z direction constrained the lower boundary displacement. The upper boundary of the model was applied with 20 MPa self-weight stress of the overlying strata.

Mohr–Coulomb yield criterion was used to judge the evolution law of stress, displacement and energy of composite floor of roadway.

### Simulation scheme

In the mining of thick coal seam working face, if the direct floor of roadway is weak or soft lithology, the floor heave problem of this kind of roadway is more serious. Therefore, most of such roadway excavation methods are bottom coal excavation, so that the bottom coal plays a role in protecting and buffering the direct bottom^14–16^.

It can be seen that the floor strength is an important factor affecting the stability of the roadway floor and the setting of the bottom coal, especially the relationship between the floor rock strength *σ*_*RC*_ and the bottom coal strength *σ*_*MC*_. Therefore, three relationships between floor rock strength *σ*_*RC*_ and bottom coal strength *σ*_*MC*_ were set: the floor rock strength *σ*_*RC*_ was greater than, equal to or less than bottom coal strength *σ*_*MC*_, namely, *σ*_*RC*_ > *σ*_*MC*_, *σ*_*RC*_ = *σ*_*MC*_, *σ*_*RC*_ < *σ*_*MC*_. The selection of floor rock parameters referred to the ‘MT 553-1996 Classification standard of working face floor in gently inclined coal seam’, etc. The specific floor lithology parameters were shown in Table 2^35,36^.

**Table 2:** Physical and mechanical parameters of different strength floors.

Floor strength	Bulk modulus/GPa	Shear modulus/GPa	Cohesion force/MPa	Friction angle/°	Tension strength/MPa
*σ*_*RC*_ < *σ*_*MC*_	2.41	1.52	1.29	25	1.36
*σ*_*RC*_ = *σ*_*MC*_	2.56	1.72	1.55	26	1.58
*σ*_*RC*_ > *σ*_*MC*_	2.74	1.87	1.70	28	1.87

**Figure 6:**
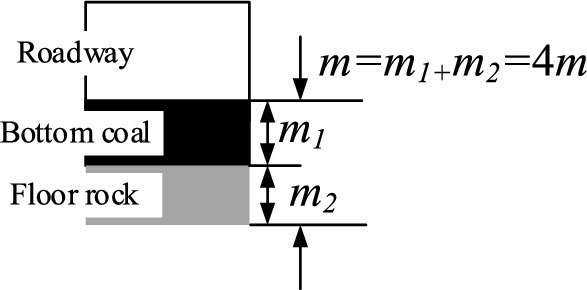
Relationship between composition and thickness of composite floor.

As one of the most directly measurable properties of bottom coal in roadway, the thickness of bottom coal has an important influence on the mechanism of bottom coal. The thickness of bottom coal will directly affect the proportion of coal-rock structure of roadway floor, and then affect the construction of floor impact model and the formation process of impact initiation. Therefore, the bottom coal thickness *m*_*1*_ of the roadway was set to 0 m, 0.5 m, 1 m, 1.5 m, 2 m, 2.5 m and 3 m. After the bottom coal is retained, the roadway floor forms a coal-rock composite floor composed of bottom coal and floor rock. Considering the proportion of coal-rock structure of roadway floor and the existing drilling detection data, the thickness *m* of coal-rock composite floor was 4 m. According to the thickness of the bottom coal *m*_*1*_ and the thickness of the composite floor *m*, the thickness of the floor rock *m*_*2*_ is set, that is, *m* = *m*_*1*_ + *m*_*2*_. As shown in Fig. 6.

**Figure 7:**
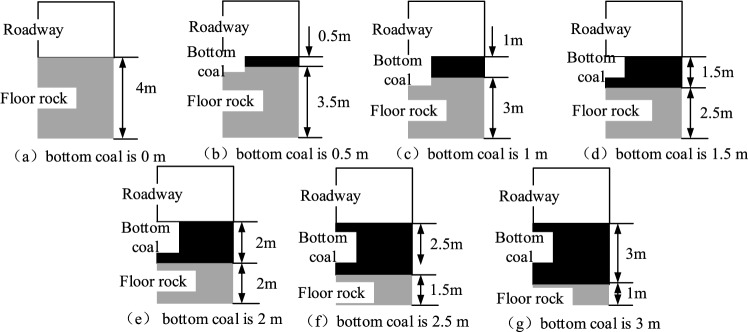
Numerical simulation scheme.

Considering the floor strength and the bottom coal thickness, 21 numerical simulation schemes were designed. The specific numerical simulation scheme was shown in Fig. 7.

### Analysis of numerical simulation results

#### Variation law of vertical stress of mining roadway floor

**Figure 8:**
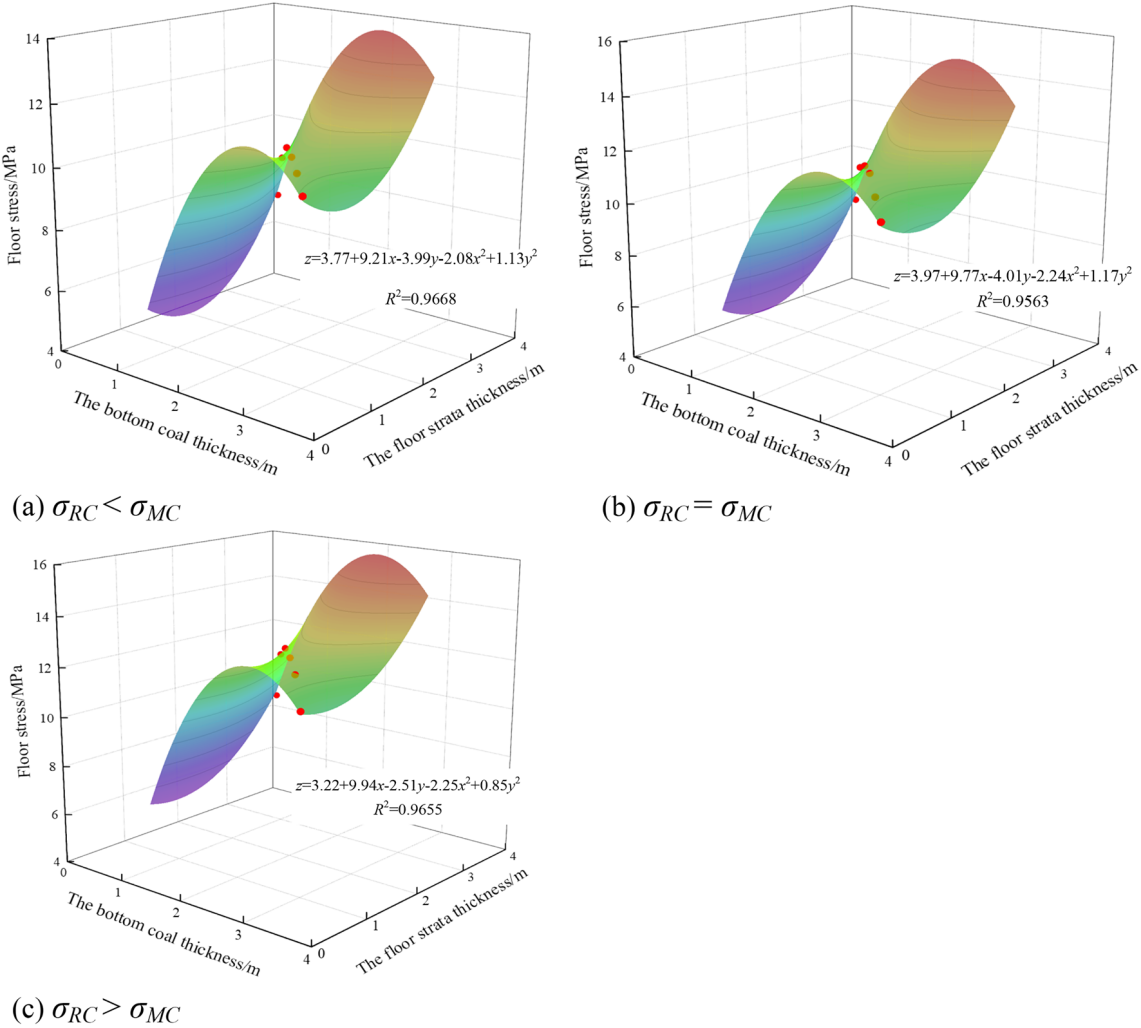
Fitting surface of floor stress, bottom coal thickness and floor strata thickness.

The stress of surrounding rock is an important factor to determine the stability of surrounding rock and the safety of chamber. The stress state of surrounding rock directly affects the stability of roadway. As the strength of roadway floor strata changed from weak to strong, the stress of mining roadway floor was fitted with the thickness of bottom coal and floor strata. The result is shown in Fig. 8.

It can be seen from Fig. 8 that the floor stress increased first and then decreased with the increase of bottom coal thickness and floor rock thickness. The maximum values of floor stress were 10.22 MPa, 11.01 MPa and 12.25 MPa when the bottom coal thickness was the same as the bottom rock thickness. The relationship between floor stress and bottom coal thickness and floor rock thickness was almost quadratic parabolic surface, and the fitting degree was greater than 95%.

Analyzing the relationship between the floor strata strength and the bottom coal strength, it can be seen that with the gradual increase of floor strength, floor stress also gradually increased. However, when *σ*_*RC*_ = *σ*_*MC*_, the increase of floor stress with the change of bottom coal thickness was the smallest, which was 34.29%. When *σ*_*RC*_ < *σ*_*MC*_, the increase was the largest, which was 36.19%.

From the perspective of stress, when the thickness of bottom coal was the same as that of floor rock, the stress of composite floor was the largest. When the thickness of the bottom coal was 0.5 m, the stress of the composite floor was the smallest. The stress of composite floor increased with the increase of floor strength. It can be seen that when the thickness of bottom coal was small and the strength of bottom strata was low, the stress of bottom strata was smaller.

#### Variation law of vertical displacement of mining roadway floor

**Figure 9:**
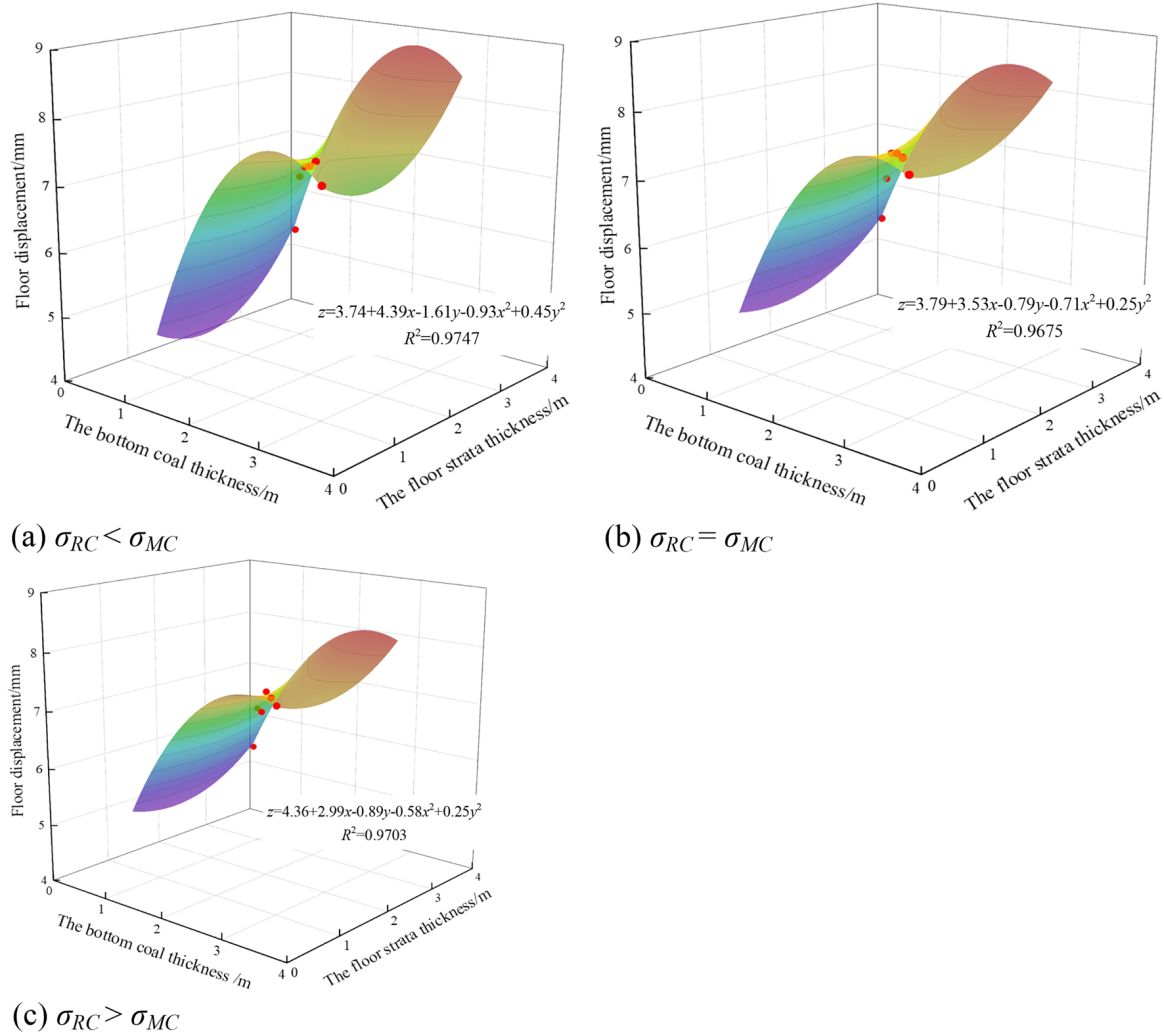
Fitting surface of floor displacement, bottom coal thickness and floor strata thickness.

The deformation of surrounding rock of roadway is one of the important indexes to measure the stability of roadway. It is an important reference for judging the existing support effect, maintaining the stability of roadway, guiding the design of on-site support and ensuring the safety production of coal mine. As the strength of the floor rock changed from weak to strong, the floor displacement of the mining roadway was fitted with the bottom coal thickness and the floor rock thickness. The result is shown in Fig. 9.

It can be seen from Fig. 9 that the displacement of roadway floor increased first and then decreased with the increase of bottom coal thickness and floor rock thickness. When the bottom coal thickness was 2.5 m, the floor displacement reached the maximum value, and the maximum displacement was 7.53 mm, 7.50 mm and 7.49 mm respectively. The relationship between floor displacement and bottom coal thickness and floor rock thickness was almost quadratic parabolic surface, and the fitting degree was greater than 95%.

Different from the floor stress, the floor displacement decreased gradually with the increase of the floor strength. When *σ*_*RC*_ = *σ*_*MC*_, the increase of floor displacement with the change of bottom coal thickness was the smallest, which was 33.61%. When *σ*_*RC*_ < *σ*_*MC*_, the increase was the largest, which was 36.66%.

According to the analysis of floor displacement, when the thickness of bottom coal was 2.5 m, the displacement of composite floor reached the maximum. When the thickness of the bottom coal is 0.5 m, the displacement of the composite floor was the smallest. As the strength of the floor rock increased, the displacement of the composite floor gradually decreased. It can be seen that when the thickness of the bottom coal was small and the strength of the floor rock was large, the displacement of the floor was smaller.

#### Variation law of elastic strain energy of surrounding rock of roadway

**Figure 10:**
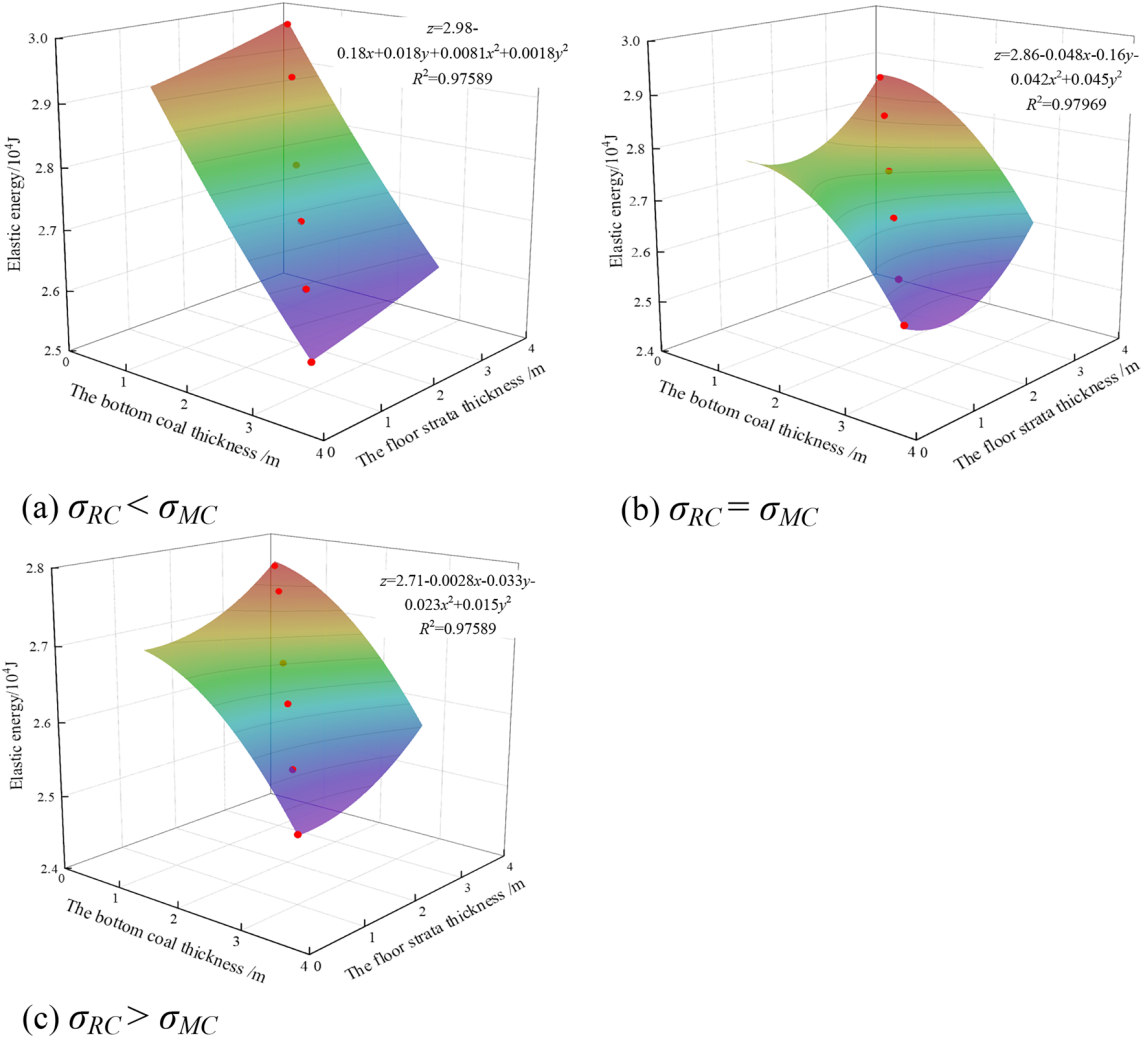
Fitting surface of floor elastic energy, bottom coal thickness and floor strata thickness.

The accumulation and release of elastic strain energy are closely related to the failure process of rock. The elastic strain energy can be used to further analyze the mechanical response and energy transformation mechanism of surrounding rock. It provides scientific basis for the design of roadway construction, support and maintenance. At the same time, the elastic strain energy accumulated in coal and rock mass is the prerequisite for the occurrence of rock burst. Understanding the accumulation degree and release law of elastic energy can effectively predict the occurrence trend and potential risk of rock burst. As the strength of the floor rock changed from weak to strong, the elastic energy of the roadway floor was fitted with the bottom coal thickness and the floor rock thickness. The result is shown in Fig. 10.

It can be seen from Fig. 10 that the elastic strain energy of roadway floor gradually decreased with the increase of bottom coal thickness, and the minimum values were 2.54 × 10^4^ J, 2.52 × 10^4^ J and 2.49 × 10^4^ J, respectively. Moreover, with the increase of floor strength, the decrease of elastic strain energy with the change of bottom coal thickness became smaller. When *σ*_*RC*_ < *σ*_*MC*_, the amplitude was the largest, which was 14.58%. When *σ*_*RC*_ > *σ*_*MC*_, the amplitude was the smallest, which was 9.85%.

By analyzing the energy law of the floor, it could be found that when the bottom coal thickness was 3 m, the energy accumulated in the composite floor was the least. When the bottom coal thickness was 0.5 m, the energy accumulated in the composite floor was the most, but the energy accumulated in the floor was less than that when the bottom coal was 0 m. It can be seen that the retention of bottom coal could effectively reduce the accumulation of energy in the roadway floor and reduce the risk of rock burst.

#### Determination of reasonable bottom coal thickness in thick coal seam roadway

It can be seen from the stress, displacement and energy variation characteristics of the composite floor that as the floor strength increased, the composite floor stress increased, and the displacement and the energy decreased. Comparing and analyzing the stress, displacement and energy of the floor with and without the bottom coal, it could be seen that the stress, displacement and energy of the floor with the bottom coal was smaller than that without the bottom coal. This is because the bottom coal plays a buffering role here, which can effectively protect the floor from damage. Further analysis of the influence of bottom coal thickness and floor rock thickness on the composite floor showed that when the bottom coal thickness was the same as the floor rock thickness, the composite floor stress was the largest. When the bottom coal thickness was greater than the floor rock thickness, the composite floor displacement was the largest. Therefore, it can be considered that when the bottom coal thickness was less than the floor rock thickness, it was a more reasonable thickness of the bottom coal. In addition, the composite floor energy decreased with the increase of bottom coal thickness.

Therefore, considering the numerical simulation results of stress, displacement and energy, the safe thickness of bottom coal in thick coal seam mining is 1 m. Under this bottom coal thickness, the surrounding rock of the roadway, especially the floor, will be in a state of low stress and small deformation, and the surrounding rock of the roadway is relatively safe as a whole.

#### Safety evaluation process of bottom coal thickness in thick coal seam roadway

**Figure 11:**
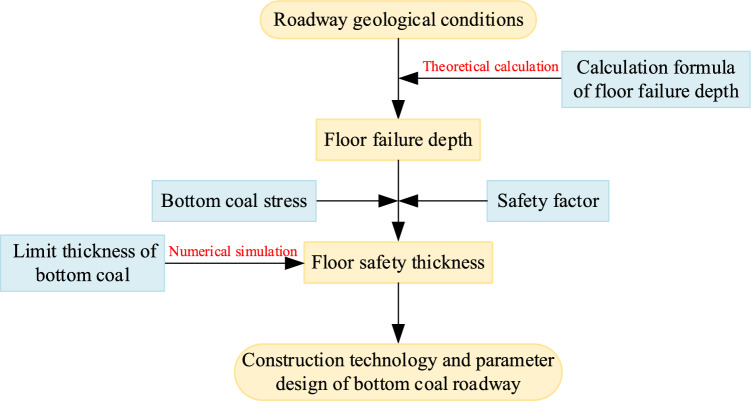
Safety evaluation process of bottom coal thickness in roadway of thick coal seam.

According to the above research and analysis, the thickness of the bottom coal in the thick coal seam roadway is related to the ultimate failure depth of the floor. On the basis of considering the safety factor and the stress of bottom coal, the calculation method of safe thickness of bottom coal in thick coal seam roadway was established. Combined with the numerical simulation method, the evaluation process of the safety thickness of the bottom coal in the thick coal seam was proposed, as shown in Fig. 11. The evaluation process of safety thickness of bottom coal in detail is as follows:

Firstly, the calculation results of the ultimate failure depth of the bottom coal roadway in thick coal seam are obtained.

Secondly, the force and safety factor of bottom coal are determined according to field measurement.

Thirdly, carry out numerical simulation analysis.

Fourthly, the safety thickness of bottom coal is given according to the results of numerical simulation.

Fifth, determine the construction technology and parameters of the bottom coal roadway.

## Discussion

The existing research data show that the coal seam of most floor burst mines is thicker, and the bottom coal with different thickness will be left in the excavation of mining roadway. Due to the large difference in physical and mechanical properties between the bottom coal and the floor rock, the remaining bottom coal part will be used as the accumulation and release area of impact energy. It will have the most direct and significant impact on the impact initiation of the floor^37–43^. As one of the main factors inducing floor rock burst, the mechanical properties of bottom coal have a great influence on the initiation of floor rock burst, such as bottom coal thickness, bottom coal strength and floor strength^44–50^. Therefore, this manuscript studied the safety evaluation of bottom coal thickness in thick coal seam roadway, and analyzed the stress and energy law of surrounding rock under different floor strength and different bottom coal thickness. The results showed that the stress of coal-rock composite floor increased with the increase of floor rock strength, and the displacement and accumulated energy of composite floor decreased gradually.

In order to study the influence of bottom coal thickness on floor rock burst, many experts and scholars have carried out research through theoretical analysis, numerical simulation and similar simulation^51,52^. The existing research results show that with the increase of the bottom coal thickness, the risk of floor impact gradually increases, but the increase gradient gradually decreases. When the thickness of the bottom coal increases to 2 m, the influence of the bottom coal thickness on the impact risk of the bottom coal reaches the minimum. Since then, the increase of bottom coal thickness has no obvious superposition effect on the impact risk of floor^22,23^. In this manuscript, through theoretical analysis and numerical simulation, it is found that when the bottom coal thickness is 1 m, the stress and deformation of roadway surrounding rock are small. The stability of roadway surrounding rock is good, which is conducive to roadway maintenance. It is a reasonable and safe thickness of bottom coal.

Some experts and scholars have found that with the increase of the anti-failure ability of the floor rock, the influence range of mining gradually decreases. And the energy accumulation range of the floor rock is negatively correlated with its own bearing capacity^53,54^. The results of this manuscript are similar to the above research results. With the increasing of floor strength, the elastic energy size and distribution range in floor decreased gradually, and the deformation and failure range of floor decreased. The results of this study have a good guiding significance for the field engineering practice of roadway bottom coal retention in thick coal seam face. It is expected to provide reference and help for related underground engineering such as roadway support in thick coal seam face.

In this manuscript, the safety of bottom coal thickness in thick coal seam roadway was systematically studied, and some valuable conclusions were obtained. However, the influence of coal pillar width, buried depth and other factors on the bottom coal retention of thick coal seam roadway has not been known. A lot of theoretical analysis, numerical simulation and engineering comparison research should be carried out in the future work. It provides a reliable theoretical support for excavation and support design of roadway in thick coal seam face.

## Conclusion

Through theoretical analysis and numerical simulation, this study systematically analyzed the stress, displacement evolution law and energy accumulation law of the roadway floor under different floor strength and bottom coal thickness. The following conclusions are obtained:

1. Aiming at the mining roadway of thick coal seam, considering the setting of bottom coal in roadway, the mechanical model of composite beam of coal-rock composite floor was established. The stress concentration area of composite floor is distributed in coal seam or rock stratum with large elastic modulus. The failure depth of floor increases with the increase of the force, and the floor heave will occur after the failure reaches the limit depth.
2. With the increase of floor strength, the stress of coal-rock composite floor also increases gradually, and the displacement and energy of composite floor decrease gradually. When the floor rock strength is equal to the bottom coal strength, the increase of floor stress with the change of bottom coal thickness is the smallest, which is 34.29%. When the floor rock strength is less than the bottom coal strength, the increase is the largest, which is 36.19%. When the floor rock strength is equal to the bottom coal strength, the increase of floor displacement with the change of bottom coal thickness is the smallest, which is 33.61%. When the floor rock strength is less than the bottom coal strength, the increase is the largest, which is 36.66%. When the floor rock strength is less than the bottom coal strength, the increase of the elastic strain energy of floor with the change of bottom coal thickness is the largest, which is 14.58%. When the floor rock strength is greater than the bottom coal strength, the increase is the smallest, which is 9.85%.
3. With the increase of bottom coal thickness, the stress and displacement of coal-rock composite floor increase first and then decrease, and reach the maximum when the bottom coal thickness is 2 m and 2.5 m respectively. The elastic strain energy of roadway floor decreases with the increase of bottom coal thickness. Based on the comprehensive analysis of the numerical simulation results of stress, displacement and energy, it is considered that the safe thickness of bottom coal in the bottom coal roadway of thick coal seam mining is about 1 m.
4. The safety evaluation process of bottom coal thickness in thick coal roadway was put forward. The main methods are as follows: a. calculate the failure limit depth of composite floor; b. determine the force and safety factor of bottom coal; c. numerical simulation analysis; d. get the safe thickness of coal; e. determine the roadway construction technology and parameters.

## Data Availability

The original contributions presented in the study are included in the article/supplementary material. Further inquiries can be directed to the corresponding author. The data used to support the findings of this research are included within the paper.
